# Erdheim-Chester Disease and Small Lymphocytic Lymphoma Collision Tumour Presenting as a Perirenal Mass

**DOI:** 10.1155/2020/3081824

**Published:** 2020-04-14

**Authors:** Antonio Maietta, Maryam Razmpoosh

**Affiliations:** Centre Hospitalier de l'université de Montréal (CHUM), 1100, rue Sanguinet, 7th Floor, F Pavillon, (Québec), Montréal, Canada H2X 0C1

## Abstract

**Background:**

Erdheim-Chester disease is a rare histiocytic neoplasm associated with MAPK pathway mutations. Disease manifestation is variable often involving many different organs, mainly bone, retroperitoneum, the heart, and the central nervous system. Histological findings include foamy histiocytes in a fibrous stroma with scattered inflammatory infiltrate. Histiocytes are CD68 positive and S100 negative. *Case Report*. We report a case of Erdheim-Chester disease associated with small lymphocytic lymphoma presenting as a perirenal mass with a review of the recent literature.

**Conclusions:**

Erdheim-Chester disease rarely can be associated with other cancers, namely myeloid neoplasms. We report a case of Erdheim-Chester disease presenting with small lymphocytic lymphoma as a perirenal mass. The association of Erdheim-Chester disease with lymphoproliferative disorders needs to be elucidated.

## 1. Introduction

Erdheim-Chester disease (ECD) is a rare non-Langerhans form of histiocytosis, first described in 1930 by Jakob Erdheim's apprentice, William Chester [1]. ECD has seen a noticeable rise in new cases over the past decade as a result of better recognition of this condition. To date, roughly 500 cases have been reported in the literature. Its origin has been debated as to whether it is a reactive versus neoplastic condition, with the latter being favored in recent years [2]. The diagnosis is mainly based on histological findings, characterized as a diffuse infiltration of bone by CD68+ and CD1a- foamy histiocytes, associated with dense fibrosis, lymphocytes, plasma cells, and Touton giant cells, with or without histiocytic infiltration of extraskeletal tissue [3, 4]. In the patients identified with ECD, there is usually at least one extraskeletal site involved, which can occur in any part of the body, most frequently being cardiovascular, retroperitoneal, pulmonary, and CNS [5]. The diagnosis requires a pathologic evaluation interpreted within the appropriate clinical context. To the best of our knowledge, we report the first case of ECD occurring simultaneously with a small lymphocytic lymphoma (SLL). We will explore its clinical presentation, the characteristics of the biopsy, and literature review.

## 2. Case Presentation

**Figure 1:**
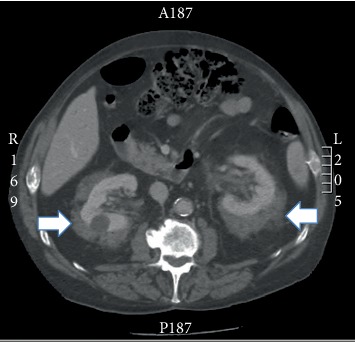
Abdominal CT scan showing symmetrical, bilateral perirenal rind-like lesions (arrows) measuring 2.5 cm in thickness, so-called hairy kidney sign.

We report the case of an 81-year-old male patient, with a previous history of postinfectious glomerulonephritis, two coronary bypass surgeries, aortic valve replacement, cholecystectomy, benign prostatic hyperplasia, and a right hemicolectomy for colon cancer. During the routine postoperation follow-up for his colon cancer, the patient indicated that he had new nonspecific abdominal pain. After a thorough clinical history yielded negative results, the patient was sent for an abdominal pelvic CT scan where the identification of rind-like bilateral perirenal masses by the radiologist resulted in the presumptive diagnosis of Erdheim-Chester disease (Figure 1). Two core biopsies of the perirenal mass were obtained. A cerebral scan and bone scan were also performed. The latter showed bilateral sclerotic lesions of all long bones from his arms and legs. The cerebral scan was negative.

**Figure 2:**
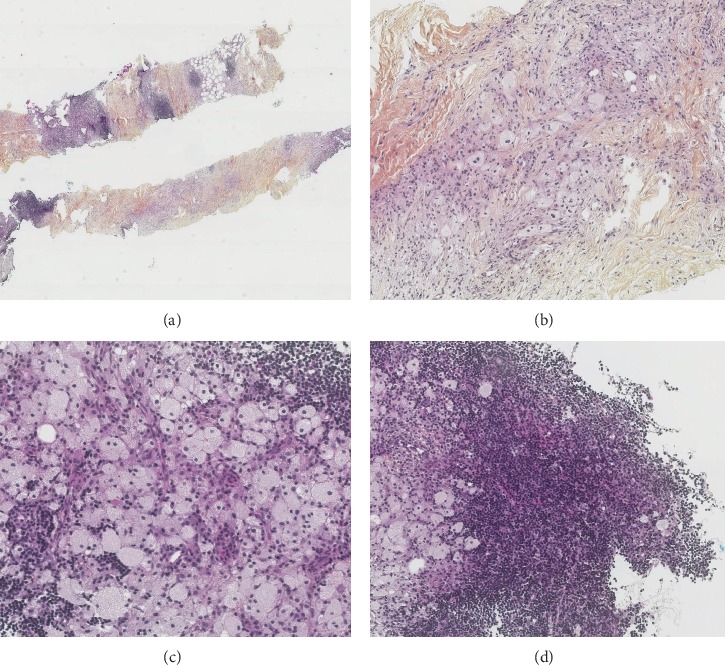
Needle biopsy of perirenal lesion showing (a) collision tumor of ECD with low grade B cell lymphoma; (b) foamy histiocytes, fibrous stroma, and scattered inflammatory cells of ECD; (c) foamy histiocytes admixed with small monomorphous lymphocytes; and (d) small lymphocytic lymphoma.

**Figure 3:**
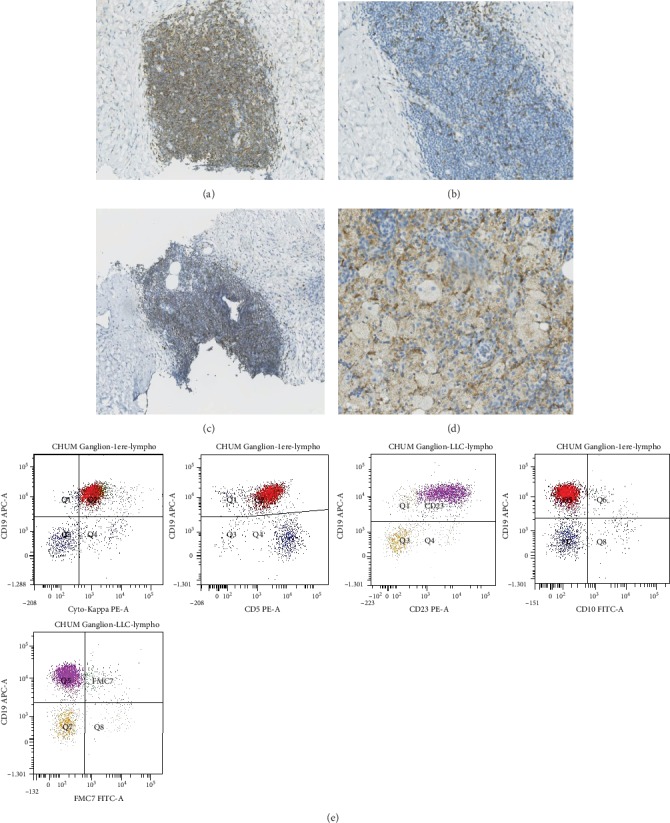
Immunohistochemical findings of perirenal mass. (a) CD79a, (b) CD3, (c) CD5, (d) CD68. (e) Flow cytometric findings of perirenal mass typical of SLL (CD19+, Kappa+, CD5+, CD23+, CD10-, and FMC7-).

The biopsy of the perirenal mass showed diffuse tissue infiltration by sheets of foamy histiocytes, interspersed with mononuclear inflammatory cells in a fibroadipose background. An atypical lymphoid infiltrate with interstitial and nodular architecture accompanied the histiocytosis (Figure 2). Immunohistochemical analysis was conducted for antibodies specific to CD20, CD79a, CD3, CD5, CD43, CD23, Bcl1, CD68, CD1a, and protein S100; the histiocytes were positive for CD68 and negative for CD1a and S100, confirming the radiologically proposed diagnosis of ECD. The small monomorphic lymphocytic population was positive for CD20, CD79a, CD5, CD43, and CD23 and negative for CD10 and Bcl1, consistent with the diagnosis of small lymphocytic lymphoma. A sample of the biopsy was sent for flow cytometric assessment and showed monoclonal B lymphocytes with kappa light chain restriction and typical SLL phenotype (Figure 3). Molecular studies did not show exon 15 V600E mutation in BRAF gene. The patient developed mild bicytopenia, anemia, and thrombopenia of unknown cause one year following the initial diagnosis. Bone marrow biopsy was performed to investigate the cytopenias. The histological and immunohistochemical analysis of the bone marrow biopsy showed minimal 5% medullary infiltration by SLL without ECD involvement. Bone marrow aspirate showed a similar lymphocytic infiltrate with flow cytometric findings consistent with minimal bone marrow involvement by SLL (not shown). The patient did not develop significant peripheral blood lymphocytosis. The maximum peripheral blood lymphocytosis reached 2.5 X10^9^/L. Lymphocytes typical of SLL/CLL were never seen on peripheral blood smear, and blood was never assessed for flow cytometric findings. Therefore, the diagnosis of monoclonal B cell lymphocytosis was never confirmed in this case. The patient was followed closely without any treatment. He had a favorable follow-up and died five years later of unrelated causes.

## 3. Discussion

ECD is a rare form of histiocytosis. The clinical manifestations are variable. Many organ systems can be affected. Most frequently, this disease manifests with bilateral symmetrical bony lesions. Cardiac manifestations are also quite common. Perirenal masses, cerebral involvement as well as periorbital lesions are other findings associated with ECD [6–9]. The histological findings of the disease are nonspecific and include foamy histiocytes in a fibrous stroma. The multinucleated giant cells of Touton-type are commonly present. A polymorphous inflammatory infiltrate including scattered plasma cells and lymphocytes is common. These findings can easily mimic a reactive process and clinical correlation is crucial for definitive diagnosis [3, 4]. ECD must be differentiated from other neoplastic histiocytoses, particularly Langerhans cell histiocytosis (LCH) and indeterminate cell histiocytosis (ICH) and from other reactive histiocytoses such as Rosai-Dorfmann disease (RDD). Immunohistochemical findings are key in differentiating these different conditions. ECD is characterized by CD68+, CD163+, CD1a-, S100-, and Langerin- histiocytes, whereas LCH contain S100+, CD1a+, and Langerin+ histiocytes. Unlike LCH, ICH is S100+ and CD1a+, but Langerin is negative [10, 11]. RDD shows characteristic emperipolesis with S100+ histiocytes.

A recent classification of histiocytic neoplasms has been published and separates the disorders into five families based on clinical manifestations and genetic findings [10]. ECD has been classified in the L-type of histiocytosis along with LCH and IDH. Although ECD is classically referred to as a non-Langerhans cell type histiocytosis, recent genetic findings show these two entities to be closely related. Patients with ECD are at increased risk of having LCH sometimes presenting together in the same lesion. The L-type histiocytoses are characterized by mutations in the MAPK pathway, particularly mutations in BRAF. However, allele burden can be quite low, and sensitive assays are sometimes required in order to detect genetic abnormalities [9].

Rare cases have been reported in the literature of ECD presenting concomitantly with another hematological cancer [12]. Most such cases are myeloid neoplasms including myeloproliferative disorders and overlap disorders such as CMML or acute leukemia. ECD occurring together with a lymphoid neoplasm in the same patient is truly a rare phenomenon [13, 15, 16]. Our case most resembles a case of ECD presenting as a collision tumour with marginal zone lymphoma [13]. In that case report, both lesions were found simultaneously in the same lymph node and the relationship between the two neoplasms could not be determined. The cases of marginal zone lymphoma have been found in association with another type of histiocytosis, reactive crystal storing histiocytosis [14]. In this scenario, the relationship is likely one of histiocytes reacting to immunoglobulin deposition without any clonal association. Sakr et al. reported a case of ECD occurring in a patient with Burkitt lymphoma [15]. In this case, the patient was treated for Burkitt lymphoma and later developed ECD. Again, a clonal relationship between the two entities could not be established. BRAF mutation was found in the ECD, but there was no mention of B cell clonality or myc rearrangement in the ECD. Other rare reports of ECD with lymphoblastic lymphoma in paediatric cases have been reported [16]. ECD associated with small lymphocytic lymphoma has never been documented before. BRAF mutation was not detected in our case. Although the two diseases manifested together in the same lesion, their genetic relationship could not be determined. In lymphoproliferative disorders, BRAF mutations are associated principally with hairy cell leukemia, a chronic lymphoproliferative disorder similar to chronic lymphocytic leukemia [17, 18]. To this date, no case of HCL with ECD has been reported. BRAF mutation occurring in CLL is a rare event [19]. The lymphoid component of our collision tumour was considered SLL, because it consisted of a mass lesion without clinical criteria meeting the definition of chronic lymphocytic leukemia (CLL). However, the patient did have mild lymphocytosis which was never characterized by flow cytometric analysis. If the lymphocytosis proved to be monoclonal, the lymphoid infiltrate would be better characterized as tissue involvement by monoclonal B cell lymphocytosis [20]. However, the clinical picture would be unusual for high count monoclonal B cell lymphocytosis, since this patient never developed CLL even after a follow-up period of more than five years.

Many examples abound in the literature of apparently divergent lineage neoplasms coexisting in the same patient. The best studied example is histiocytic sarcoma existing with low grade B cell lymphoma, notably follicular lymphoma [21–24]. In fact, every case of histiocytic sarcoma should be screened for B cell clonality studies as well as BCL2 rearrangement. Rarer cases of SLL/CLL associated with histiocytic sarcoma also occur in the literature [25, 26]. The cases of CLL occurring with haemophagocytic lymphohistiocytosis have also been described [27]. Epigenetic mechanisms as well as transcription factors involved in lineage determination have been implicated in this divergent differentiation model of a collision tumour [28]. Another well-studied example is transformation of follicular lymphoma into diffuse large B cell lymphoma. This does not appear to occur through a linear accumulation of mutations but rather through divergent differentiation of an early common precursor clone [29]. In our particular case, a chance association of ECD with fairly common age-related monoclonal B cell lymphocytosis can also be a potential explanation for the existence of a collision tumour. The exact mechanisms involved in collision tumours involving ECD and lymphoid neoplasms need to be studied.

In summary, this patient showed characteristic rind-like bilateral perirenal masses with bilateral sclerotic lesions of all long bones of his arms and legs. Histologically, the perirenal mass consisted of a diffuse infiltration by non-Langerhans type foamy histiocytes leading to the diagnosis of Erdheim-Chester disease. In addition, a low-grade B cell lymphoproliferative process was admixed with the lesion and proven to be a small lymphocytic lymphoma by immunohistochemical studies and flow cytometric analysis. To our knowledge, this is the first case of a collision tumour involving ECD and SLL ever reported.

## Abbreviations

ECD: Erdheim-Chester disease
SLL: Small lymphocytic lymphoma
LCH: Langerhans cell histiocytosis
ICH: Indeterminate cell histiocytosis
RDD: Rosai-Dorfmann disease
CMML: Chronic myelomonocytic leukemia
CLL: Chronic lymphocytic leukemia.
